# Lipoprotein(a): Insights for the Practicing Clinician

**DOI:** 10.3390/jcm11133673

**Published:** 2022-06-25

**Authors:** Pyotr Telyuk, David Austin, Ahai Luvai, Azfar Zaman

**Affiliations:** 1 Academic Cardiovascular Unit, The James Cook University Hospital, Middlesbrough TS4 3BW, UK; david.austin@nhs.net; 2 Department of Clinical Biochemistry, The Newcastle upon Tyne Hospitals NHS Foundation Trust, Newcastle upon Tyne NE1 6ND, UK; ahailuvai@nhs.net; 3 Department of Cardiology, The Newcastle upon Tyne Hospitals NHS Foundation Trust, Newcastle upon Tyne NE1 6ND, UK; azfar.zaman@nhs.net

**Keywords:** lipoprotein(a), cardiovascular disease, thrombogenicity

## Abstract

Following the discovery of the Lipoprotein(a) (Lp(a)) molecule by Kare Berg in 1963, many physiological and pathological properties of this particle remain to be fully understood. Multiple population-based studies have demonstrated a correlation between elevated Lp(a) levels and the incidence of cardiovascular disease. Data extrapolated from the Copenhagen City Heart and ASTRONOMER studies also demonstrated the link between Lp(a) levels and the incidence and rate of progression of calcific aortic stenosis. Interest in Lp(a) has increased in recent years, partly due to new emerging therapies that can specifically reduce serum Lp(a) concentrations. Given the strong correlation between Lp(a) and CV disease from epidemiological studies, several international guidelines have also been updated to advocate Lp(a) testing in specific population groups. This review aims to highlight the importance of the role of Lp(a) in cardiovascular disease and discusses the potential of novel therapies in patients with elevated Lp(a) levels.

## 1. Introduction

Elevated Lp(a) plasma concentrations are known to be associated with ischemic heart disease and calcific aortic stenosis [1]. This evidence has been generated from multiple population-based studies and meta-analyses involving large patient cohorts [2,3,4]. Lp(a) levels are mostly heritable and determined by single-nucleotide variants of the Lp(a) gene. Given the strong genetic links and evidence for raised Lp(a) in premature ischemic heart disease, understanding the pathological properties of this molecule is important. Moreover, optimal management of CV risks including LDL-C only has a minimal impact on levels of Lp(a), for which there is no available therapy at present. We discuss the major recent developments in therapeutics in this field, although, to date, specific treatments targeting Lp(a) levels are undergoing clinical trials. In this review, we highlight the pathogenic role of Lp(a) and discuss available management strategies and new developments.

## 2. Lipoprotein(a) Genetic Inheritance and Molecular Structure

**Figure 1:**
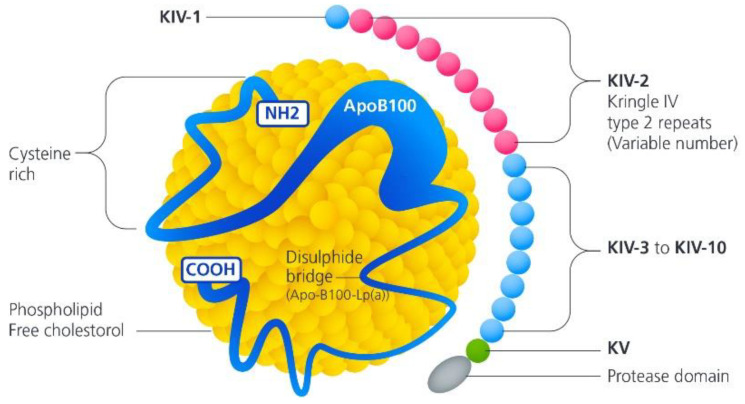
Schematic representation of Lp(a) particle. Lp(a) is composed of apo(a) covalently linked to apo(b100).

Lp(a) is a genetically determined, liver-derived molecule with a unique structure (Figure 1). It is a spherical complex similar in structure to low-density lipoprotein (LDL). Despite the structural similarity between LDL and Lp(a), the synthesis of one molecule is completely independent of the other. In vitro studies suggest that the assembly of Lp(a) occurs extracellularly, at the hepatocyte surface or in the plasma. On the other hand, studies in human hepatocytes indicate Lp(a) assembly within hepatocyte cells. The exact location of synthesis, however, is unclear [5]. Another significant difference between LDL and the Lp(a) molecule is the presence of a second protein called apolipoprotein (a) (apo(a)), which is covalently linked to apolipoprotein (b) through a disulfide bond [6].

Lp(a) is encoded by the Lp(a) gene on chromosome 6, which evolved from the plasminogen gene. Apo(a) is composed of highly glycosylated, three-dimensional heavy-chain-peptide structures called Kringles and an inactive protease domain similar to plasminogen. The protease domain of Apo(a) is catalytically inactive due to the replacement of serine amino acid by arginine in the active site, and, therefore, apo(a) does not exert any fibrinolytic activity exhibited by plasminogen [7]. The catabolism of the Lp(a) molecule is not well understood. Animal studies suggest that Lp(a) clearance predominantly occurs within the liver and, to a lesser degree, by the kidneys, with multiple receptors playing a role in Lp(a) catabolism, including lipoprotein receptors, toll-like and scavenger receptors, lectins, and plasminogen receptors [5]. 

Kringle domains play an important role in blood coagulation. The Lp(a) molecule contains a copy of the plasminogen Kringle V domain and 10 types of Kringle IV domains (1–10) with Kringle IV type 2 repeated in multiple copies. The number of Kringle IV type 2 repeats determines the isoform size and mass of Lp(a) [8]. The higher the molecular mass, the slower the rate of apo(a) production, and, therefore, individuals with a lower molecular mass of apo(a) have higher serum Lp(a) levels [9]. Patients with a high concentration of the smaller Lp(a) isoform and, therefore, fewer copies of Kringle IV type 2 repeats exhibit high cardiovascular risk. Given its specific and unique structure, the pathogenic role of Lp(a) has been thought to promote atherosclerosis (due to its similarity with LDL) and thrombosis due to its structural similarity with plasminogen [10]. Moreover, investigators have also reported a role of Lp(a) in angiogenesis, wound healing, and tumor growth.

## 3. Measurement of Lipoprotein(a)

Given the Lp(a) size heterogeneity due to variable numbers of Kringle IV type 2 copies, there has been a major challenge in determining the accurate measurement of Lp(a). Some antibodies used against Apo(a) cross-react with KIV-2 repeats and, therefore, overestimate Lp(a) concentration in individuals with large isoforms and underestimate the concentration in those with smaller isoforms. It is, therefore, possible that the lack of association of Lp(a) and CVD risk in some older studies was driven by the use of isoform-sensitive assays, which provided inconsistent measurements of Lp(a). Isoform-insensitive methods calibrated in nmol/L and traceable to WHO/IFCC reference material provided a more consistent Lp(a) measurement [11].

Lp(a) measurements can be expressed in molecular mass (mg/dL) or molar concentration (nmol/L). Mass includes all particle constituents, while molar units are more indicative of particle numbers. Due to heterogeneity and the presence of two isoform sizes in most individuals, standardization using a single calibrant material is unreliable. Conversion between mass and molar units is inherently inaccurate and should be avoided. 

Approximately 1 in 5 Europeans have elevated Lp(a) concentrations, which is defined as a level greater than 120 nmol/L or greater than 50 mg/dL [12]. There are no gender differences in Lp(a) concentrations; however, an ethnic difference has been observed with White and Asian people having lower Lp(a) levels with higher isoform size, whereas people of African and Hispanic ethnicities have higher Lp(a) levels with smaller isoform size. A UK consensus statement has proposed graded cardiovascular disease risk categories based on Lp(a) centiles as follows [9]:

- 32–90 nmol/L—minor risk of CV disease;
- 90–200 nmol/L—moderate risk;
- 200–400 nmol/L—high risk;
- >400 nmol/L—very high risk.

Several international guidelines have updated the statements on Lp(a) measurement in population cohorts. Lipid guidelines published by the European Society of Cardiology recommend at least a single lifetime measurement of Lp(a) in the general population [13]. The American College of Cardiology guidelines give a relative indication for Lp(a) measurement in patients with an established family history of premature CVD [14]. The Canadian Cardiovascular guidelines have also recently updated their recommendation on Lp(a) measurement. Previously, Lp(a) testing was encouraged in individuals with intermediate Framingham risk scores, whereas, now, the consensus appears to be that Lp(a) measurement is performed at least once in a patient’s lifetime [15]. NICE guidelines on cardiovascular disease: risk assessment and reduction, including lipid modification, were last updated in 2016 and do not refer to Lp(a) measurement. 

## 4. Lipoprotein(a) and Cardiovascular Disease

Several epidemiological studies have reported associations between elevated Lp(a) levels and cardiovascular disease (Table 1). One UK-based observational study of over 450,000 participants with a median follow-up of over 11 years demonstrated a linear rise in the risk of atherosclerotic cardiovascular disease with higher Lp(a) concentrations [16]. Further observational data show a relationship between higher Lp(a) levels and the incidence of myocardial infarction. The study involved over 12,000 patients stratified by ethnicity and adjusted for age and sex. The association of elevated Lp(a) concentration with new MI was independent of cardiovascular risk factors including diabetes, hypertension, and smoking. This observational study has also demonstrated an inverse association of isoform size and Lp(a) concentration, indicating a lower risk of MI with higher isoform size [17]. The observational data suggest the estimated risk of acute MI in patients with Lp(a) > 50 mg/dL to be three-fold [18]. A large meta-analysis of over 126,000 individuals demonstrated an increased incidence of coronary heart disease and stroke (16% and 10%, respectively) in patients with elevated Lp(a) levels [5]. Beyond the association with coronary atherothrombosis, data from the Copenhagen City Heart Study and the Copenhagen General Population Study, which observed over 77,000 patients for up to 20 years, demonstrated an increased risk of aortic valve stenosis in the population with elevated Lp(a) levels. An estimated three-fold increased risk was predicted with levels >90 mg/dL [19]. The post hoc analysis of the Astronomer trial also supported the concept of a greater rate of progression of aortic valve stenosis and the requirement for earlier intervention in subjects with elevated Lp(a) [20].

## 5. Potential Thrombogenic Role for Lp(a)

Several studies have also explored the thrombogenic role of Lp(a). It is thought that Lp(a) may play a role in thrombosis by promoting the aggregation and activation of platelets, inhibiting tissue factor pathway inhibitor (TFPI), reducing the production of plasmin, and increasing the expression of plasminogen activator inhibitor-1 (PAI-1) [21]. However, several in vitro studies reported conflicting results suggesting that Lp(a) decreases platelet activation. One mechanistic study reported a decreased fibrin clot permeability in patients with elevated Lp(a) [22]. These conflicting data suggest further studies are required to assess the effect of Lp(a) on thrombus formation. The Lp(a) thrombogenicity study aims to evaluate the effect of high Lp(a) concentration on thrombus using the ex vivo Badimon perfusion chamber [23].

Given the potential pathogenic properties of Lp(a), patients with elevated levels may suffer from a greater myocardial injury at the time of myocardial infarction. One retrospective analysis suggested a probable higher rate of periprocedural myocardial injury in patients with elevated Lp(a) undergoing elective percutaneous coronary intervention [24]. The exact mechanisms for this are unknown but may be a combination of the effects of Lp(a) described above. A study evaluating the effects of Lp(a) on thrombotic and restenotic events following coronary stent implantation included over 2000 patients with successful PCI and found no association of angiographic restenosis or stent thrombosis with Lp(a), although one limiting factor was the relatively short (6 months) duration of the follow-up after the original procedure [25]. The optical coherence tomography (OCT) sub-study by Niccoli et al. suggested an increased prevalence of high-risk plaque features such as thin-cap fibroatheroma in ACS patients with Lp(a) levels greater than 30 mg/dL [26].

Despite the potential effect of Lp(a) on plaque morphology and cardiovascular disease, the data on mortality are conflicting. A recent review of myocardial infarction patients by Wohlfahrt et al. did not show a link between high concentrations of Lp(a) and increased risk of total mortality [27]. An increased mortality in this observational study was seen in the very-low-Lp(a) group, with the potential explanation for this correlation being the role of Lp(a) in tissue healing. This hypothesis, however, requires further research.

## 6. Lp(a) and Universal Testing

Given that most guidelines now encourage Lp(a) testing predominantly in higher-risk cohorts, some concerns have been raised due to the lack of evidence that Lp(a) testing leads to improved patient outcomes [28]. This concern is compounded by the lack of available specific therapies proven to reduce Lp(a) levels. Currently, the mainstay of management is aimed at reducing the overall cardiovascular risk factor profile and controlling overall dyslipidemia.

## 7. Therapeutic Developments

Several potential therapies have been thought to reduce Lp(a) concentration or target the pathogenic properties of this molecule (Table 2). The use of aspirin therapy for primary prevention in this patient group is controversial given previously published extensive data demonstrating elevated bleeding risks, particularly in older patients [29]. Conversely, data extrapolated from a retrospective analysis of a Women’s Health study found that carriers of an Lp(a) variant benefited from aspirin therapy [30].

The role of statin therapy on Lp(a) concentration is equally controversial. A meta-analysis published in the European Heart Journal including over 5000 patients demonstrated an increase in Lp(a) concentration amongst participants receiving statin therapy. The mechanism by which statins may increase Lp(a) concentration is not completely understood [31]. On the other hand, data extrapolated from the JUPITER study involving over 9000 patients demonstrated no median change in Lp(a) with rosuvastatin treatment. Importantly, a significant reduction in the incidence of cardiovascular disease was demonstrated in statin trials [32].

Niacin is another potential therapy evaluated for the treatment of patients with elevated Lp(a), with studies demonstrating a modest decrease in Lp(a) concentrations [33] but not associated with a reduction in major adverse cardiovascular events (MACEs). A similar effect was demonstrated with cholesterol ester transferase protein (CETP) inhibitors. The ACCELERATE trial demonstrated Lp(a) reduction with the use of CETP inhibitors but without a decrease in MACE [34].

The use of PCSK9 inhibitors in the FOURIER and ODYSSEY studies confirmed a modest reduction in Lp(a) concentration of up to 15% [35]. A non-randomized study from Berlin published in 2021 demonstrated a 36% reduction in Lp(a) concentration following one month of treatment with higher doses of Alirocumab or Evolocumab. Interestingly, there was a negative correlation between the molecular size of Apo(a) and the absolute reduction in Lp(a) induced by PCSK9 inhibitors [36]. Another retrospective analysis that looked at Lp(a) levels following the addition of PCSK9 inhibitors to niacin also found a modest reduction of 15% in Lp(a) concentration [37]. ORION 10 and ORION 11 trials assessed the efficacy of inclisiran, a small interfering RNA agent that reduces the hepatic synthesis of PCSK9. Trials demonstrated an overall Lp(a) reduction by around 20% [38].

Currently, there are no approved pharmacological therapies for patients with elevated Lp(a). In recent years, antisense oligonucleotides that target hepatic Lp(a) messenger RNA have been the subject of intense research. A Phase 2, randomized, double-blind, placebo-controlled clinical trial reported in 2021 studied 286 patients with established cardiovascular disease and Lp(a) levels of at least 60 mg/dL. Patients were randomized into five dose-dependent groups to receive a second-generation antisense oligonucleotide (AKCEA-APO(a)-L_Rx_) versus placebo. The primary endpoint was the percentage change in Lp(a) levels from baseline to 6 months of receiving IMP. Secondary outcomes included the percentage change in LDL cholesterol, percentage change in apo(b) levels, and percentage of patients with Lp(a) levels equal to or below 50 mg/dL. The study demonstrated a significant reduction in Lp(a) concentration across all dose groups (between 35% and 80% reduction depending on the dose received) following 6 months of treatment with the hepatocyte-directed antisense oligonucleotide. The effect was seen within the first month of treatment and reached maximal effect by week 16. The percentage of patients with Lp(a) levels of 50 mg/dL or less was 98% in the group receiving 20 mg of IMP weekly. The LDL-C change was present but relatively small, taking into account that the majority of patients received statin therapy and 20% of patients were on a PCSK9 inhibitor. Adverse events were commonly reported in both IMP and placebo groups. Importantly, the incidence of adverse events or serious adverse events did not show a dose-dependent pattern. The most common side-effects were myalgia and malaise, but no marked changes in platelet count, or renal or liver function tests were seen [39]. 

Given the positive outcome of this phase 2 trial, the phase 3, randomized, double-blind, placebo-controlled HORIZON trial aims to assess the effect of second-generation antisense oligonucleotide TQJ320 on the incidence of major adverse cardiovascular events in patients with elevated Lp(a). The study plans to enroll 7680 participants with a medical history of cardiovascular disease and Lp(a) levels of at least 70 mg/dL. The primary outcome of the study is the combined incidence of cardiovascular death, non-fatal MI, nonfatal stroke, and urgent coronary revascularization requiring hospitalization. The HORIZON trial will expect to complete recruitment in 2022, and the clinical community will keenly await the results of the first phase III trial targeting elevated Lp(a) [40].

## 8. Conclusions

Multiple epidemiologic studies have confirmed an association between the pathological role of Lp(a) in atherothrombotic cardiovascular disease and aortic valve stenosis. Universal testing, however, remains a subject of discussion given multiple challenges in the measurements due to isoform-sensitive assays and the lack of available therapies, demonstrating a positive effect on CVD outcomes in patients with elevated Lp(a). Although the Lp(a) structural homology to plasminogen may potentially lead to increased thrombogenicity and decreased fibrin clot degradation, conflicting study results indicate that further mechanistic studies are required. More recently, the discovery of antisense oligonucleotides that target hepatic Lp(a) messenger RNA offers an opportunity to significantly reduce serum Lp(a) levels. The ongoing HORIZON trial will inform us about the efficacy of TQJ230 on cardiovascular events in high-cardiovascular-risk patients with high Lp(a) when added to standard lipid-lowering therapies.

## Figures and Tables

**Table 1:** Epidemiological studies suggesting a causal role of Lp(a) in CVD.

Epidemiological Studies	Patient Cohort	Population	Outcome	Results
The Copenhagen City Heart study	10,855	General population	Registry-based CV outcomes	Increased risk of MI and AVS
The Copenhagen General Population study	66,877	General population	Incidence of AVS	3-fold increased risk of AVS with Lp(a) > 90 mg/dL
Danesh J et al.: meta-analysis of 27 prospective studies	5436	General population	Incidence of CHD	Increased incidence of CHD
Erqou et al.: Lipoprotein(a) concentration and the risk of coronary disease, a meta-analysis of 36 prospective studies	126,634	General population	Incidence of CHD and CVA	Increased association of Lp(a) with CHD and CVA
Pare et al.: Lipoprotein(a) levels and the risk of MI among 7 ethnic groups, INTERHEART study	12,943	General population	Incidence of MI	Increased risk of MI

**Table 2:** Effects of lipid-lowering agents on Lp(a) concentration.

Drug/Intervention	Lp(a) Level Reduction	CV Risk Reduction
Aspirin	N/A	Yes
Statin therapy	Conflicting results suggesting potential increase in Lp(a) concentration	Yes
Lipoprotein apheresis	>50%	Yes
Niacin	20–25%	No
Bempedoic acid	No	Yes
Monoclonal antibodies to PCSK9 inhibitors	20–30%	Yes
Inclisiran	20%	No
Antisense oligonucleotides	80%	No (trials are ongoing)

## Data Availability

No new data were created or analyzed in this study. Data sharing is not applicable to this article.

## Abbreviations

Apo(a)	Apolipoprotein (a)
AVS	Aortic Valve Stenosis
CHD	Coronary Heart Disease
CV	Cardiovascular
CVA	Cerebrovascular Accident
LDL-C	Low Density Lipoprotein Cholesterol
Lp(a)	Lipoprotein a
MI	Myocardial Infarction
